# HER2, NF-*κ*B, and SATB1 Expression Patterns in Gastric Cancer and Their Correlation with Clinical and Pathological Parameters

**DOI:** 10.1155/2019/6315936

**Published:** 2019-10-14

**Authors:** Marta Smolińska, Dariusz Grzanka, Paulina Antosik, Anna Kasperska, Izabela Neska-Długosz, Jakub Jóźwicki, Anna Klimaszewska-Wiśniewska

**Affiliations:** Department of Clinical Pathomorphology, Faculty of Medicine, Collegium Medicum in Bydgoszcz, Nicolaus Copernicus University in Torun, Poland

## Abstract

Gastric cancer (GC) is currently recognized as one of the most common and fatal tumor worldwide. The identification of novel biomarkers in relation to clinical information as well as extending the knowledge on a multiple crosstalk between various oncogenic pathways implicated in GC carcinogenesis seems pivotal to limit the disease-associated mortality. Therefore, we assessed the expression of HER2, NF-*κ*B, and SATB1 in a total of 104 gastric adenocarcinomas and 30 normal gastric samples and correlated the expression patterns with each other and with some clinicopathological variables. Protein expression was examined by immunohistochemistry (IHC) on tissue microarrays (TMAs), and fluorescence *in situ* hybridization (FISH) was employed to detect HER2 amplification. In the studied group, HER2 and SATB1 were found to be overexpressed in gastric cancer tissue in comparison to normal gastric mucosa. The expression status of the former protein was seen to differ according to some clinicopathological features, but without statistical significance, whereas the expression of the latter was not importantly associated with any of them. In turn, the NF-*κ*B protein level was significantly related to the presence of lymph node metastasis. HER2 expression was not significantly correlated with that of other proteins, but a positive correlation was found between the expression of SATB1 and NF-*κ*B. Further studies with a larger group of patients combined with *in vitro* mechanistic experiments are required to fully elucidate the role and relationship of HER2, NF-*κ*B, and SATB1 expression in gastric cancer progression. However, to the best of our knowledge, this study is the first look at a simultaneous evaluation of these three markers in the samples of gastric cancer patients.

## 1. Introduction

Gastric cancer (GC) is the fifth most common malignancy and the third leading cause of cancer-related deaths globally, following lung and liver cancer [1]. Each year, approximately 990 000 people throughout the world are diagnosed with gastric cancer, of whom about 738 000 die annually, accounting for 8% of new cancer cases and 10% of cancer deaths [2–4]. Although the incidence and mortality is geographically varied and highly prevalent in Asia, particularly in China (approximately 24 per 100 000 cases in men and 9.8 per 100 000 cases in women), this is almost half of the total gastric cases in the world [5, 6]. Most new cases and deaths occur in East Asian countries, but other high incidence areas of gastric cancer are South America and Eastern Europe, while lower rates are in North America, Africa, and Northern Europe [2, 3]. Histologically, there are three main types of gastric cancer: diffuse-type, intestinal-type, and mixed-type adenocarcinoma. Diffuse-type gastric cancer consists of individually infiltrating neoplastic cells throughout the gastric mucosa [7]. Pathogenesis of the intestinal-type gastric adenocarcinoma is a multistep progression and the transition from normal mucosa to chronic superficial gastritis, atrophic to intestinal metaplasia, finally to dysplasia and adenocarcinoma [8]. There is evidence that the intestinal type of gastric cancer is related to chronic *Helicobacter pylori* infection [9]. Mixed-type gastric adenocarcinoma comprises histologically non-homogenous mixtures of diffuse and intestinal carcinomas.

*Helicobacter pylori* infection is probably the strongest risk factor of gastric cancer and plays a critical role in gastric cancer pathogenesis. According to the World Health Organization, *H. pylori* is recognized as a class I carcinogen associated with gastric cancer. More than 80% of gastric cancer may be associated with signaling pathways caused by *H. Pylori* infection [10, 11]. The nuclear factor-kappa B- (NF-*κ*B-) dependent pathway interacts with *H. pylori* peptidoglycans through nucleotide-binding and oligomerization domain 1 (Nod1), leading to the activation of proinflammatory responses—IL-8 or *β*-defensin-2 [12]. The activation of the NF-*κ*B pathway controls the expression of the coding genes, including cytokines, chemokines, pro- and antiapoptotic factors, angiogenesis regulator vascular endothelial growth factor (VEGF), and matrix metalloproteinases (MMPs). NF-*κ*B is constitutively active in many types of cancer and can exert a variety of protumorigenic functions. Gastric cancer is preceded by the multistep carcinogenesis process, including chronic inflammation (the initial step), atrophy, metaplasia, and dysplasia. Chronic infection caused by *H. pylori* accounts for majority of cases of non-cardia gastric cancer. *H. pylori* infection activates NF-*κ*B-dependent chemokine production in epithelial cells of gastric mucosa [13]. *H. pylori* utilizes many different mechanisms for the induction of proinflammatory cytokines. It has been shown that the bacterial products are particularly important for the activation of NF-*κ*B [14]. There are also a lot of other pathways associated with gastric carcinogenesis.

The human epidermal growth factor receptor (HER) family involves four tyrosine kinase receptors with similar structure—HER1, HER2, HER3, and HER4, also called ErbB1, ErbB2, ErbB3, and ErbB4, respectively. HER-family tyrosine kinases activate downstream pathways involved in a regulation of key cellular functions, and they are expressed in epithelial, mesenchymal, and neuronal cells as well as their cellular progenitors [15, 16]. Specifically, activation of these receptors by ligand binding initiates a complex cascade of intracellular events that begins with autophosphorylation and activation of tyrosine kinase domain and further involves initiation of several downstream signaling pathways, such as the phosphatidylinositol-3-kinase (PI3K)/Akt/mammalian target of rapamycin (mTOR) pathway, the central Ras/Raf/mitogen-activated protein kinases (MAPK) pathway, and the phospholipase C*γ* (PLC*γ*) pathway, among others [17]. These signaling pathways affect numerous target proteins and transcription factors leading to the alterations of various cellular functions, such as proliferation, differentiation, migration, adhesion, angiogenesis, apoptosis, and survival [18]. Therefore, it should not be surprising that mutations and overexpression of HER family members have found to be associated with uncontrolled cell proliferation and metastasis and thereby implicated in the development and progression of many tumors, including gastric cancer [15]. In the latter, the overexpression of HER2 was first described in 1986. The *HER-2/neu* (*EERB2*) gene is located in chromosome 17 (17q21) and encodes a transmembrane tyrosine kinase receptor—p185 [19].

Special AT-rich binding protein 1 (SATB1) is a protein encoded by the *SATB1* human gene located on chromosome 3p23 and is mainly related to the development of thymus cells [20, 21]. SATB1 is a well-known cell type-specific nuclear matrix protein, which selectively binds special AT-rich sequence of matrix attachment regions (MARs). In a double-stranded DNA, through the presence of altered sugar-phosphate backbone, SATB1 recognizes AT-rich elements. Binding to a base-unpairing regions (BURs), at least in part, leads to folding of higher-order chromatin loop domains—that is the reason why SATB1 is called global chromatin organizer [22, 23]. SATB1 is engaged in chromatin reconstruction processes, histone acetylation, and methylation, and through these functions, it enables the regulation of multiple genes [24]. SATB1, as a nuclear factor, is involved in the regulation of the expression of more than 1000 genes [22]. Many recent studies have shown that SATB1 is highly expressed in several cancers and correlated with aggressiveness, poor survival, and clinicopathological properties. Additionally, it plays a major role in the process of carcinogenesis, invasion, progression, and metastasis of cancer [25–30]. In the case of some tumors, it has been proven that SATB1 is involved in the development of chemoresistance [31, 32]. The role of SATB1 is dependent on the type of tumor and other potential factors. The specific function of SATB1 still remains not fully known, especially in the context of mechanisms underlying the development of malignant phenotype of cancer cells. Due to the complex changes acquired in a multistage process of stomach carcinogenesis, the tumor itself is heterogeneous and exhibits many genetic changes. The genetic and epigenetic alterations act at different stages of carcinogenesis, leading to dysregulation of various genes. Finding novel, potential biomarkers not only may broaden our knowledge about the genetic basis of stomach cancer but also may help with estimating the risk of the occurrence of this cancer.

The main aim of this research was the immunohistochemical assessment of the expression of the selected proteins, with a potential (NF-*κ*B, SATB1) or proven (HER2) role in the pathogenesis of gastric cancer, both in the tumor tissue and in the normal gastric mucosa. This study also includes the analysis of the expression status of these proteins in relation to each other and to clinicopathological features. To the best of our knowledge, no studies have been carried out on the simultaneous evaluation of these three markers in gastric cancer samples.

## 2. Material and Methods

### 2.1. Material

This research was performed on tissue specimens from 104 patients with gastric adenocarcinoma who underwent gastrectomy at the Department of Transplantology and General Surgery, Collegium Medicum in Bydgoszcz, Nicolaus Copernicus University in Torun (Poland) between 2007 and 2015. For the purpose of gathering a suitable study group, all tumors were histopathologically reexamined, including the confirmation of diagnosis, the number of lymph nodes with metastasis reclassification based on the standardized TNM 7^th^ classification by the American Joint Committee on Cancer (AJCC) [33]. The control group consisted of 30 normal gastric mucosa tissues from the patients who underwent endoscopy between 2016 and 2017.

### 2.2. Ethics Statement

The present study was approved by the Bioethical Commission of Collegium Medicum in Bydgoszcz of the Nicolaus Copernicus University in Torun, Poland (issue: *KB 76/2018*).

### 2.3. Methods

Immunohistochemical studies on formalin-fixed, paraffin-embedded (FFPE) specimens were conducted at the Department of Clinical Pathomorphology, Collegium Medicum in Bydgoszcz, Nicolaus Copernicus University in Torun, Poland.

#### 2.3.1. Tissue Microarrays

Histological reevaluation of hematoxylin-eosin- (H&E-) stained slides enables choosing duplicated 2 mm cores containing representative tumor areas with at least 80% of tumor cells. Selected archival paraffin blocks (donor blocks) were reembedded with the use of paraffin mixed with wax to the form of dimensions 37 × 24 × 7.5 mm. Tissue microarrays (TMAs) were obtained by transferring representative tissue fragments from donor blocks using an automated tissue arrayer (TMA Master; 3DHISTECH, Budapest, Hungary) into recipient block. Additionally, in order to verify tumor cells, HE staining was performed from TMA blocks. Next, paraffin-embedded TMA block was cut into 3-4 *μ*m thick sections, using a manual rotary microtome (Accu-Cut, Sakura Finetek, Torrance, CA, USA). The prepared sections were then placed on extra adhesive slides (Superfrost Plus; Menzel-Glaser, Braunschweig, Germany). The primary rabbit monoclonal anti-HER2/neu (4B5) antibody (Ventana Medical Systems, Tucson, AZ, USA; FDA-approved clone for the assessment of HER2 status), rabbit polyclonal anti-NF-*κ*B p65 (ab16502) antibody (Abcam, Great Britain), and rabbit monoclonal anti-SATB1 (EPR3895) (ab92307) (Abcam, Great Britain) antibody were used to test the expression of HER2, NF-*κ*B, and SATB1 proteins. Standardization of IHC procedure was performed using a series of positive and negative control reactions on FFPE tissue sections. Positive control was performed on a tissue model in which the presence of the antigen was indicated on the basis of antibody data sheet and reference sources (The Human Protein Atlas: http://www.proteinatlas.org) [34]. SATB1-positive control reaction was performed on a tonsil tissue, showing the nuclear expression of the protein, NF-*κ*B on lymph node showing cytoplasmic expression, and HER2 on breast cancer tissue showing membranous expression. Additionally, negative control reactions were performed by replacing a primary antibody with a 1% bovine serum albumin (BSA) diluted in phosphate-buffered saline (PBS).

#### 2.3.2. Immunohistochemical Staining of HER2

The immunohistochemical staining was performed using an automated system BenchMark GX Platform (Ventana Medical Systems, Tucson, AZ, USA) with rabbit monoclonal anti-HER2/neu (4B5) antibody (Ventana Medical Systems, Tucson, AZ, USA). The reaction was performed using the visualization system (UltraView DAB Detection Kit; Ventana Medical Systems, Tucson, AZ, USA). Additionally, stained preparations were dehydrated, cleared in xylenes, and mounted with Shandon Consul Mount (Thermo Scientific, Waltham, USA).

#### 2.3.3. Immunohistochemical Staining of NF-*κ*B and SATB1

Prepared slides with tissue sections were deparaffinized and rehydrated. In the first step, antigens were retrieved using a high-pH buffer (Dako, Agilent Technologies, USA) at 95-98°C for 20 min in PT Link (Dako, USA). Then, to block the endogenous peroxidase activity as well as the nonspecific binding sites, the preparations were incubated with 3% H_2_O_2_ (10 min at room temperature (RT)) and 3% BSA (15 min at RT), respectively. The incubation with the primary rabbit polyclonal anti-NF-*κ*B antibody (1 : 400) and rabbit monoclonal anti-SATB1 antibody (1 : 100) was performed for 30 min at RT. The use of EnVisionFlex+ Anti-Mouse/Rabbit HRP-Labeled Polymer (Dako, Agilent Technologies) for 20 min at RT and 3,3′diaminobenzidine (DAB) enabled the localization of the antigen-antibody complex. In addition, the tissues were counterstained in Mayer's hematoxylin. Finally, tissue sections were dehydrated in ethanol of increasing concentration (from 80% to 98%), then cleared in a series of xylenes (from I to IV), and cover-slipped in a medium (Dako, Agilent Technologies, USA).

#### 2.3.4. Expression Analysis

The immunohistochemical evaluation of protein expression was performed in a blinded fashion by two independent pathologists in the light ECLIPSE E400 microscope (Nikon Instruments Europe, Amsterdam, Netherlands).

HER-2 immunostaining was scored according to a 4-tier HercepTest scoring system modified for gastric carcinoma by Hofmann et al. [35] as follows: 0—no reactivity or membranous reaction in fewer than 10% of cells, (1+)—faint complete or partial membranous reactivity in more than 10% of cells, (2+)—moderate complete or basolateral membranous reactivity in more than 10% of cells, and (3+)—strong complete or basolateral membranous reactivity in more than 10% of cells. The level of HER2 membranous expression was considered positive if IHC staining was 2+ or 3+ followed by confirmation of equivocal (2+) IHC scores with fluorescence *in situ* hybridization (FISH). In conjunction with these GC-specific scoring principles, the degree of microscopic magnification required to accurately identify membranous staining was selected based on “magnification rule” presented by Rüschoff et al. [36]. Accordingly, the visualization of IHC 1+, 2+, and 3+ scores needs high magnification (×40), medium magnification (×10-20), and low magnification (×2.5-5), respectively.

The expression of NF-*κ*B and SATB1 was analyzed at 20x original objective magnification and according to the modified Index Remmele-Stegner (IRS) scale [37], in which the percentage of positively stained cells/areas was multiplied by the intensity of staining. The scores for positive immunoreactivity were categorized as follows: (0)—less than 10% of stained cells/area; (1)—11-20% of stained cells/area; (2)—21-50% of stained cells/area; (3)—51-80% of stained cell/area; and (4)—equal or more than 81% of stained cells/area, whereas the staining intensity was evaluated using the following criteria: (0)—negative; (1)—low staining; (2)—moderate staining; and (3)—strong staining. The final staining score ranges from 0 to 12. For NF-*κ*B cytoplasmic/nuclear staining, the IHC results below or equal to 4 were considered as those without overexpression (negative); otherwise, they were defined as overexpressed (positive). In the case of nuclear SATB1 staining, the IHC scores less than or equal to 2 were classified as negative, while those greater than or equal to 3 were regarded as positive (overexpressed).

#### 2.3.5. FISH

Cases scored as 2+ were considered equivocal for HER2 protein expression, and new 4 *μ*m thickness whole-tissue sections were submitted to fluorescence *in situ* hybridization (FISH). FISH was conducted with the HER2 FISH pharmDx™ Kit (Dako, Agilent Technologies, USA) according to the manufacturer's instructions. Sections were baked overnight at 56°C, deparaffinized in three 10 min changes of xylene, and then rehydrated through three 5 min changes of 70%, 85%, and 99.8% ethanol. The slides were then reduced for 15 min in pretreatment solution at >98°C and briefly washed in 3 × PBS at RT. The slides were then incubated for 7 min in enzyme reagent solution at 37°C and washed in 3 × PBS at RT, dehydrated through 70%, 85%, and 99.8% ethanol, and allowed to air dry. After open air drying, the HER2 DNA probe kit (HER2 FISH pharmDx™ Kit, Dako, Agilent Technologies, USA), which was denatured at 82°C for 5 min, was applied onto each slide; a coverslip was added and then sealed with a coverslip sealant. After 16 h of hybridization at 45°C, the slides were washed with 65°C preheated posthybridization buffer for 10 min and dehydrated through 70%, 85%, and finally 99.8% ethanol. After air drying, the slides were counterstained with DAPI (4′,6′-diamidino-2-phenylindole) and chilled for 30 min at 4°C. Finally, the slides were observed through a fluorescence microscope (Nikon Eclipse 80i) with a ×100 oil immersion objective. The ratios of *HER2/neu* signals to CEP17 signals were calculated as follows: when the ratio was <1.8, the *HER2/neu* gene was considered nonamplified and when it was >2.2, the *HER2/neu* gene was considered to be amplified. When the ratio was between 1.8 and 2.2, signals in another 20 nuclei were counted and the HER2/CEP17 ratio in a total of 40 nuclei was determined.

#### 2.3.6. Statistical Analysis

Statistical analysis was carried out with the GraphPad Prism (version 7.01, GraphPad Software, La Jolla, CA, USA). To evaluate the differences between the expression status of SATB1, HER2, NF-*κ*B, and clinicopathologic characteristics in gastric cancer patients, the two-tailed Chi-squared test or Fisher's exact test was used. To assess the correlations between the expression status of SATB1, HER2, and NF-*κ*B, Spearman's correlation coefficient was employed. A value of *P* < 0.05 was considered to be statistically significant.

## 3. Results

### 3.1. Clinicopathological Findings

The present study included 73 male (70.2%) and 31 (29.8%) female patients with a mean age of 67.5 years (median 68, range 42-84 years). Among 104 patients, 64 (61.5%) had a positive lymph node status, whereas 40 (38.5%) were negative. Gastric carcinomas were classified according to Lauren's criteria as intestinal type in 52 (50.0%), diffuse type in 41 (39.4%), and mixed type in 11 (10.6%) cases. Histologically, they were divided into well differentiated (G1), moderately differentiated (G2), and poorly differentiated (G3). According to a histological grade, two cases (1.9%) were classified as G1, 45 (43.3%) as G2, and 57 (54.8%) as G3. Regarding the pathologic T stage, most patients were at pathologic stage T3-T4 (*n* = 65; 62.5%), 29.8% (*n* = 31) and 7.7% (*n* = 8) at stage T1-T2 and T0, respectively. Tumor localization was cardia in 33 (31.8%), fundus in 38 (36.5%), antrum in 12 (11.5%), and pylorus in 21 (20.2%) cases. In terms of tumor size, 41 (39.4%) cases were <5 cm and 63 (60.6%) were ≥5 cm. Clinicopathological features are presented in Table 1.

### 3.2. HER2 Status in Gastric Cancer and Normal Gastric Tissues: Clinicopathological Associations

**Figure 1:**
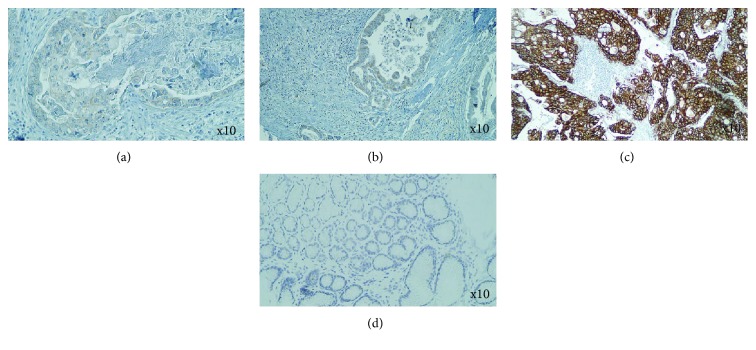
Immunohistochemical analysis of HER2 expression in gastric adenocarcinoma (primary magnification ×10). (a) Negative expression of HER2. (b) Score 2+ basolateral membrane staining for HER2. (c) Positive strong reaction (3+) basolateral membrane staining for HER2. (d) Control tissue of normal gastric mucosa.

**Figure 2:**
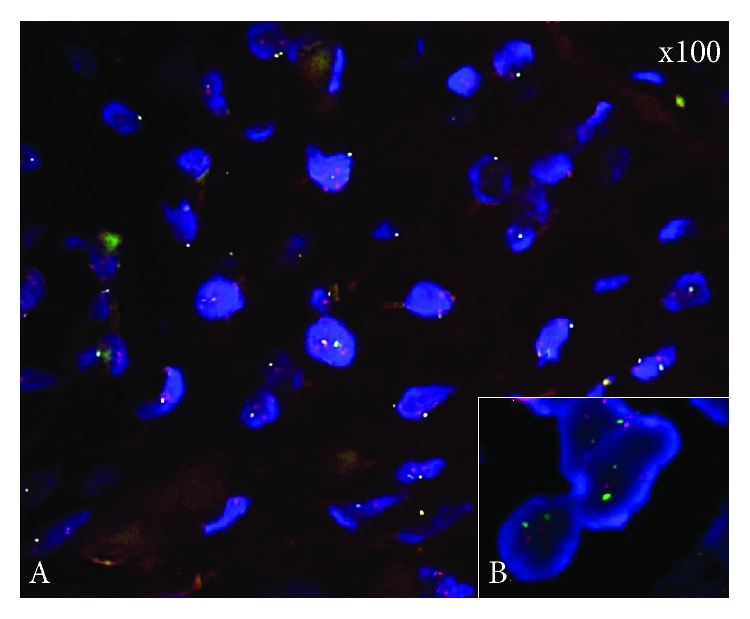
Representative cases of FISH analysis in gastric cancer (a, b). Case with HER2 amplification using FISH analysis. Green signals refer to the probe of Chr. 17 centromere, while red signals are the target probe for HER2 (primary magnification, ×100).

Immunohistochemical analysis of the membranous expression of HER2 revealed that out of a total of 104 GC cases, 10 (9.62%) were scored 3+, another 10 (9.62%) had a score of 2+, 11 (10.58%) were labeled 1+, and the remaining 73 (70.19%) were marked as 0 (Figure 1). All cases yielding equivocal (2+) IHC results were subjected to FISH assay in order to determine the final HER2 status. FISH was positive in 100% of IHC score 2+ cases (*n* = 10/10; mean HER2/CEP17 ratio per nucleus 3.97, 95% confidence interval (CI) 3.48-4.44) (Figure 2). Overall in gastric carcinomas, HER2 was found to be overexpressed (IHC score 3+ or IHC score 2+ and FISH positive) in 19.23% of total cases. The HER2 expression level in GC tissues was significantly higher when compared to normal gastric mucosa tissues, in which negative membranous expression was observed (*P* < 0.0001, *n* = 30/30, 100%) (Figure 1). However, cytoplasmic and nuclear immunoreactivity was seen in the latter cells; nevertheless, both of these immunostained areas were not taken into account for the evaluation of the HER2 expression status.

The relationship between HER2 expression and GC clinicopathological features is summarized in Table 2. Almost significantly different HER2 positivity rate was detected when comparing younger and older age groups (33.33% vs. 14.29%, respectively, *P* = 0.05). Surprisingly, the median age at diagnosis tended to decrease according to the HER2 expression status (negative, 70.5 years vs. positive, 61.5 years). However, none of the HER2-positive patients were younger than 51 years of age. Furthermore, HER2 overexpression was more frequent in patients with lymph node metastasis than in those free of lymph node metastasis (23.44% vs. 12.50%), but this association was not statistically significant (*P* = 0.21). The HER2-positive rate occurred in 22.22% of intestinal-type, 14.63% of diffuse-type, and 22.22% of mixed-type tumors. No significant correlation was found between the positive expression of HER2 and the depth of invasion, gender, tumor location, tumor size, differentiation degree, and just mentioned Lauren classification (*P* > 0.05).

### 3.3. NF-*κ*B Expression in Gastric Cancer and Normal Gastric Tissues: Clinicopathological Associations

**Figure 3:**
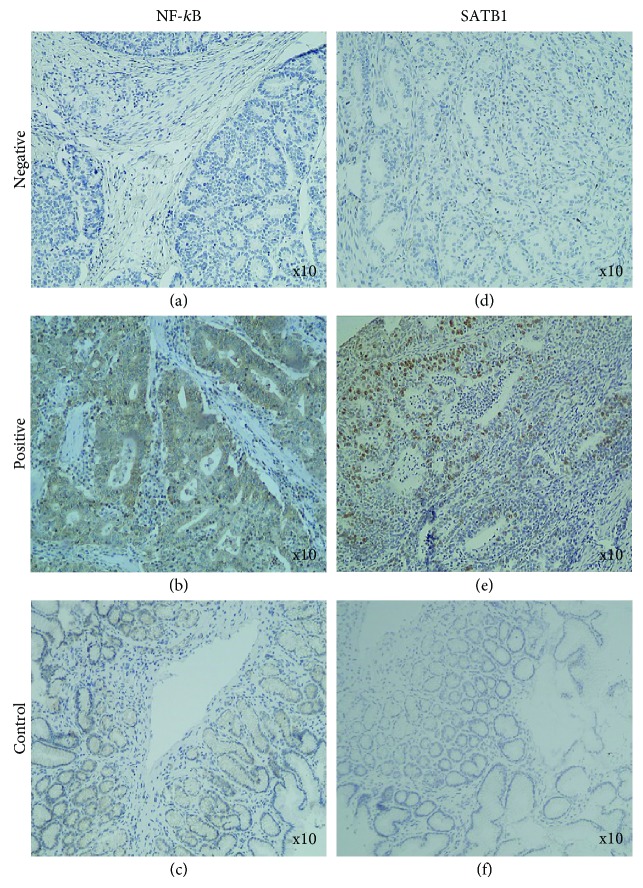
Immunohistochemical analysis of NF-*κ*B and SATB1 expression in gastric cancer tissues (primary magnification ×10). (a) Negative expression of NF-*κ*B. (b) Strong positive (3+) nuclear and cytoplasmic staining for NF-*κ*B. (c) NF-*κ*B control tissue of normal gastric mucosa. (d) Negative expression of SATB1. (e) Strong positive (3+) nuclear staining for SATB1. (f) SATB1 control tissue of normal gastric mucosa.

The IHC staining of NF-*κ*B was detected in the nuclear and cytoplasmic compartments of gastric cancer cells, while it was restricted to the cytoplasm of normal gastric mucosal cells (Figure 3). Positive immunoreactivity was found in 37 (35.58%) GC cases, whereas the remaining 67 (64.42%) were negative. In turn, NF-*κ*B was positively expressed in 17 (56.67%) of normal gastric samples and the other 13 (43.33%) had negative expression. However, the differences in the expression level of NF-*κ*B between control and GC tissues were statistically insignificant (*P* = 0.60).

The positive NF-*κ*B expression was significantly associated with the presence of lymph node metastasis (*P* = 0.04). According to Lauren classification, NF-*κ*B positivity was more common in the intestinal histological type (44.44%) than in the diffuse type (21.95%), and this correlation was estimated to be significant (*P* = 0.03). However, when taking into account an equal frequency of NF-*κ*B positivity in the intestinal and mixed types of gastric carcinoma (44.44%), then the association between NF-*κ*B status and Lauren classification was only marginally significant (*P* = 0.07). Furthermore, moderately differentiated tumors showed a higher prevalence of NF-*κ*B overexpression (46.67%) than the well (0%) and poorly differentiated ones (28.07%), but without statistical significance (*P* = 0.17). Likewise, NF-*κ*B positivity was more frequently detected in male (39.73%) than in female (25.81%) although this trend was not statistically significant (*P* = 0.19). In turn, the expression status of NF-*κ*B was not associated with age, pT stage, tumor location, or tumor size (*P* > 0.05). The relationship between NF-*κ*B expression and GC clinicopathological features is summarized in Table 2.

### 3.4. SATB1 Expression in Gastric Cancer and Normal Gastric Tissues: Clinicopathological Associations

SATB1 was expressed in the nucleus of gastric cancer cells, and the positive rate was 30.77% (*n* = 32/104). The expression level of this protein in GC tissue was markedly higher compared to normal gastric mucosa where no immunoreactivity was seen (*P* < 0.0001, *n* = 0/30, 0%) (Figure 3). The expression status of STAB1 was not associated with any clinicopathological data listed in Table 2 (*P* > 0.05).

### 3.5. Correlation between the Expression of HER2, NF-*κ*B, and SATB1 in Gastric Cancer

**Figure 4:**
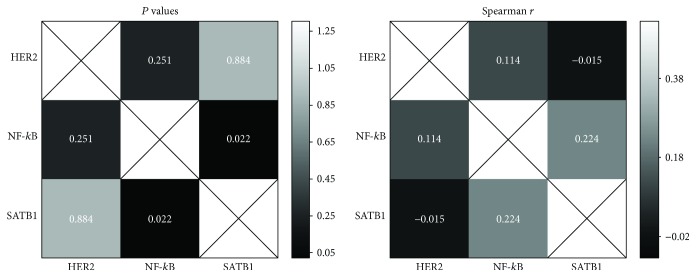
Correlation between HER2, NF-*κ*B, and SATB1 expression in gastric cancer tissues. Correlation values are presented in a heat map (Spearman correlation test).

A weak positive and significant association was confirmed between the expression of SATB1 and NF-*κ*B (*P* = 0.02, *r* = 0.22, Spearman coefficient). In the entire cohort, the expression of HER2 was not significantly correlated with the expression of SATB1 and NF-*κ*B (Figure 4).

## 4. Discussion

Biomarkers play an important and still increasing role in the screening, diagnosis, and management of cancer patients. Currently, the unique validated predictive biomarker for response to targeted therapy in gastric carcinomas is HER2. However, its prognostic significance as well as a positivity rate in this tumor type remains a matter of controversy in the literature. To date, numerous studies have examined the association of HER2 status with a prognosis of GC patients, and some of them have failed to find it [38, 39], whereas a few have shown HER2 overexpression as a favorable prognostic factor [40]. However, the vast majority of studies have found HER2 positivity to be associated with a poor clinical outcome and thus to serve as a negative prognostic factor [41, 42]. Although, due to a lack of survival data of the cohort, our research cannot take a position on the former issue, we provide a valuable information on the prevalence of HER2 overexpression in the group of 104 GC patients of Polish origin, making the assessment according to the updated guidelines for HER2 testing in this disease entity. As a result, we are able to join the discussion on the latter controversial issue regarding HER2 and GC—a large discrepancy in the incidence rate of HER2 overexpression or amplification across studies. Indeed, the earlier series of IHC- and FISH-based research have revealed a wide range of HER2 positivity rates in GC samples, from 7% to 34.0% [43, 44] and 7% to 43% [44–46], respectively. There is currently a lot of understanding that the most important reasons for the discrepancies in the reported HER2-positive rates include the use of non-standarized assays with different antibody clones and the application of various methods of evaluation and scoring schemes with different cutoff points and interpretation criteria for stained slides. In the case of gastric cancer, the determination of a new set of immunoscoring principles has been particularly important, due to the inherent biological differences between gastric and breast cancer, especially tumor heterogeneity (focal staining) and the occurrence of basolateral or lateral membrane staining [47]. Therefore, it should be taken into account that many of the previously reported results were obtained using the breast cancer HER2 testing and scoring criteria or were performed with nonvalidated assays, and as such, they must be interpreted with a great caution [48]. The present research followed the modified HER2 immunoscoring system devised for gastric cancer by Hofmann et al. [35] as well as the current recommended testing algorithm, in which immunohistochemistry should be used as the initial testing method and FISH or silver *in situ* hybridization (SISH) should be employed to retest samples with an equivocal IHC (2+) score [36, 47, 49]. Furthermore, for evaluation of membranous staining specific for the cited scoring system [35], a proper microscope magnification was applied according to “magnification rule” described by Rüschoff et al. [36, 47]. The definition of HER2 positivity included in the present study was based on the approved indication by the European Medicine Agency (EMA; IHC 3+ or IHC 2+/FISH-positive) [47], and after proceeding according to all the above-mentioned rules, the overall positivity rate of 19.23% was found in our cohort. This rate was higher than that demonstrated in the ToGA trial (16.6%)—when taking into account the applied definition of higher HER2 overexpression (IHC 2+/FISH-positive or IHC 3+), or lower (22.1%)—when the definition of HER2 positivity included all FISH-positive cases in addition to IHC 3+ samples, and finally, it was quite comparable (20.6%)—when only cancers of the stomach rather than both stomach tumors and those of the gastroesophageal junction (GEJ) were considered [49, 50]. The study on 78 GC samples from Polish patients has published a rate of 29.5% using the IHC 2+/3+ criterion based on the Hofmann et al. scoring system [35], as well as a rate of 30.7% using the IRS 4-12 criterion according to the Remmele and Stegner [37] immunoreactive score (IRS) modified by the authors. However, in the cited study, there were 28.1% of equivocal (2+) cases, which were not subjected to FISH analysis, and only 7.7% of 3+ cases [51]. Although the overall agreement between IHC and FISH is high [50], the concordance rate for the IHC 2+ group is frequently not satisfactory enough [50] or even very low [52, 53]. Surprisingly, a complete concordance between both techniques could be seen within our IHC 2+ group being all amplified by FISH. This may be due to the fact that in our cohort, there were only few equivocal cases (*n* = 10) and no cases of CEP17 polysomy within the counted nuclei. It has also been suggested that 4B5 antibody, which was used in the present study, yields a high correlation with *in situ* hybridization methods and better than other tested antibodies [54, 55]. On the other hand, we should bear in mind the limitation of our study, and in this context, we cannot rule out some underestimation of the prevalence of HER2 overexpression due to the use of the TMA technique rather than whole tissue sections, as in the study of Halon et al. [51]. TMA is a cost-effective and rapid method for analyzing numerous samples using a single IHC protocol, which allows to avoid experimental variability [56]. However, the obvious disadvantage is that this preparation enables the analysis of only a limited area of tumor sample; therefore, the intratumoral heterogeneity seems to be a main limitation of the use of TMAs for the evaluation of expression/amplification status in GC [57]. Indeed, in the study comparing TMA technique *vs*. whole tissue sections as well as three different antibodies, the use of 4B5 antibody on whole tissue sections was suggested to be the most accurate IHC method for assessing the HER2 expression in gastric adenocarcinoma [57]. In order to at least partially overcome this limitation, the assembly of two cores, retrieved from microscopically selected (distinct) representative areas of each tumor, into a single recipient TMA block was done.

In addition to the evaluation of HER2 status in our cohort, its association with the selected clinicopathological parameters was also examined. In many recent studies, HER2 overexpression in GC patients has shown to be associated with some clinicopathological features, such as age, gender, Lauren classification, histological differentiation, TNM classification, localization, and tumor size. However, the literature is conflicting at this point, and other reports did not show any significant associations between these parameters [58–61], which is also in accordance with our findings. Nonetheless, we demonstrated an almost significant HER2 correlation with patients' age. The patients with HER2-positive tumors were younger, but none of HER2-positive patients were under 51, and the age median was 61.5 years. The consensus of majority of the reports on GC [62], including the ToGA trial [50], is that Lauren's intestinal subtype is a pathological feature most invariably associated with HER2 overexpression. According to our data, the correlation of HER2 overexpression and Lauren classification was not statistically significant, however, as expected, was consistent with the previous reports stating that a positive expression of HER2 was more frequent in the intestinal type of gastric cancer (22.22%) than in the diffuse-type gastric carcinoma (14.63%). Simultaneously, there was an equal rate of HER2 positivity in the intestinal-type (22.22%) and mixed-type (22.22%) GC, which may be attributable to the fact that our mixed-type cases are predominantly consisted of intestinal histological component (mixed-predominantly intestinal type). In addition, we revealed more frequent overexpression of HER2 in patients with lymph node metastasis than in those without lymph node metastasis (23.44% *vs.* 12.50%)—the observation which has been confirmed by other authors [60]; nevertheless, in our study, this relation did not reach a statistical significance. This paragraph can be concluded that the variation in this aspect of HER2 and GC studies seems to be related not only to well-known intratumoral staining heterogeneity resulting in discrepancies in HER2 positivity rate, and thus divergences in associations of HER2 status with clinicopathological parameters, but also to the ethnicity, different sample sets (including sample size), and uneven distribution of the clinicopathological data.

It is also worthy to mention that in our control group, in which no membranous HER2 immunoreactivity was observed, the cytoplasmic and nuclear staining patterns could be seen, which may be due to a non-specific background, described previously for 4B5 antibody in GC samples [54] as well as a few areas of gastric mucosal metaplasia or dysplasia. According to the recent recommendations [36], both of these immunostained areas were not taken into account for the evaluation of the HER2 expression status.

Another proposed biomarker for GC patients is NF-*κ*B, the transcription factor that interacts with multiple upstream and downstream signaling pathways, and it is thought to play an important role in the invasion, angiogenesis, and metastasis in various neoplasms, including gastric cancer [63]. However, the special role of NF-*κ*B in the pathogenesis and progression of gastric adenocarcinoma remains unclear and controversial. As in the case of HER2, conflicting data have been reported on the association of NF-*κ*B expression with prognosis of GC patients—there are those demonstrating a correlation with poor survival [63–65] or better survival [66]. The same can be seen for the reports examining the NF-*κ*B positivity rate (a wide range from 18% to 78.3% [67]) and the relationship of the NF-*κ*B expression status with clinicopathological features of GC patients—from the studies showing a significant relation with some of the traditional clinicopathological parameters, like age, gender, T stage, tumor size, tumor location, histologic grade, Lauren classification, and nodal status [68–70], to those demonstrating no association with any of these features [65]. In our study, the cytoplasmic and nuclear immunoreactivity of NF-*κ*B was found to be positive in 35.58% of GC cases and 56.66% of normal tissue samples, but in the case of the latter ones, the immunostaining was restricted to the cytoplasm, possibly pointing to a known role of nuclear NF-*κ*B in cell proliferation. Similar staining pattern and NF-*κ*B positivity rate have been reported in GC samples by Sheng et al. [67] and Yamanaka et al. [64], respectively. In our cohort, NF-*κ*B overexpression was significantly more frequent in GCs of the intestinal histological type (44.44%) than in those of the diffuse type (21.95%), and a similar association has been found by Levidou et al. [63]. However, when taking into account our data set with an equal frequency of NF-*κ*B positivity in the intestinal and mixed types of gastric carcinoma (44.44%), then the association between NF-*κ*B status and Lauren classification was only marginally significant. As we mentioned above for HER2, our mixed-type cases have predominantly intestinal-type histology, which probably also explains an equal positivity rate of NF-*κ*B in both subgroups of patients. In addition, our studies as well as those of others [66, 69] have revealed the significant association between the presence of lymph node metastases and NF-*κ*B overexpression, which might suggest the correlation between the altered expression of NF-*κ*B and aggressiveness of gastric carcinomas. Furthermore, we have found that the positive expression of NF-*κ*B more frequently occurred in male than in female (39.73% *vs*. 25.81%), as well as in moderately differentiated tumors (46.67%) than well (0%) and poorly differentiated ones (28.07%), although both these trends were not statistically significant. In the present study, no relationship was seen between NF-*κ*B positivity and other factors, like age, the depth of invasion, tumor location, and tumor size. Since it is generally accepted that in gastric cancer the depth of invasion is closely related to the presence or absence of lymph node metastasis [71], a lack of the association between NF-*κ*B expression and pT stage in our cohort is quite unexpected, given the observed correlation of this protein with the nodal status. One explanation for this disagreement may be a marked difference of case numbers in the pT group.

In the past few years, several studies have shown the function of SATB1 as a prognostic biomarker in various types of cancers, such as breast, colorectal, pancreatic, and prostate cancer and other solid tumors, including gastric cancer [26, 72–76]. The results have been disputed, especially in the aspect of clinicopathological features and prognosis. The prognostic value of SATB1 differs depending on cancer type, which is probably a result of tissue-dependent regulatory functions of SATB1 [77]. However, there are also contradictory results even in the same tumor types that the best example can be breast cancer [72, 78], suggesting that the discrepancy may reflect differences in experimental design (e.g., antibody clones, or measuring the transcript level of *SATB1* in whole tumor tissue samples *vs*. scoring the level of SATB1 protein in particular cells, with an emphasis on its subcellular location) and scoring systems and the subjectivity of the pathologists' interpretation. Specifically, the authors of the meta-analyses have pointed out that most of the recent studies had limited power to investigate the relationship between SATB1 expression and patients' clinicopathological characteristics due to the small sample sizes [79, 80]. Although the current study also suffers from the latter drawback as well as uneven number of participants in some of the analyzed subgroups, it may support relatively scarce reports regarding SATB1 and gastric cancer [76, 80–83]. Here, we found that the expression of SATB1 was higher in GC tissues compared to normal gastric mucosa, and this observation is consistent with the previous reports [76, 81]. The SATB1-positive rate was 30.77% and lower than the rates reported by Lu et al. and Cheng et al. in the Chinese population [76, 81]. We suppose that the use of the TMA technique in this field could result in the discrepancy between the positivity rates; however, some country-specific factors may also not be negligible. These reasons seem to be particularly accurate in the light of the study of Hedner et al., who have used the TMAs along with immunohistochemistry to evaluate SATB1 status in the European population of patients with adenocarcinoma in the upper gastrointestinal tract (esophagus, cardia, and stomach), and found the rate (31.18%) very similar to ours [82]. Furthermore, the above-cited reports have demonstrated that increased SATB1 expression is independently connected with worse predictions [76, 81, 82]. Additionally, in the *in vitro* study, Sun et al. have confirmed the correlation between SATB1 expression and aggressive tumor behavior and also proposed that SATB1 plays an essential role in multidrug resistance [84]. Several studies of other authors have also shown that SATB1 overexpression is associated with the features of more aggressive tumors [29, 76]; however, we did not confirm this correlation, as we found no relationship between SATB1 status and the clinicopathological data. Thus, SATB1 appears to be a potential biomarker for GC, but to answer the question of whether it could improve diagnosis, prognosis, and prediction of recurrence and treatment response in this group of patients, further studies with large cohorts are currently urgently needed.

Since each of the studied proteins have been individually implicated in the pathogenesis of GC, and because it has been proposed that they could regulate and/or participate in the overlapping signaling pathways leading to carcinogenesis, it was reasonable to examine whether these proteins may be interconnected in terms of their expression status in our set of GC samples. SATB1 is well-known for its ability to regulate the expression of as many as 1000 genes associated with cancer development and progression, and *HER-2/neu* appears to be one of these genes [72]. The studies have revealed that SATB1 may upregulate the expression of HER2 in various breast cancer cell lines and that SATB1 expression was correlated with HER2 amplification in breast cancer tissues [31, 85]. Furthermore, it has been suggested that breast cancer patients with SATB1/HER2 coexpression tended to have even worse prognosis than those with single positive expression [85]. In the present study, we found no correlation between the immunoexpression of SATB1 and HER2 in GC samples, in contrast to Yuan and Li [83], who have shown that the SATB1 mRNA level and HER2 protein expression were positively correlated in GC patients. Bearing in mind that mRNA measurements usually include cancer cells, normal cells, and tumor-associated stromal cells (and therefore they may be subjected to error [82, 86]) and that our studies as well as those of Yuan and Li [83] included only the small number of cases (104 *vs*. 60), it would be of interest to apply the IHC method to a large cohort of GC patients for a better assessment of a possible relationship between SATB1 and HER2.

Moreover, there are also studies showing the association of SATB1 with NF-*κ*B signaling. For instance, Zhang et al. have revealed that in colorectal cancer, SATB1 expression is connected with the expression of NF-*κ*B, cyclin D1, MMP2, and PCNA [74]. Furthermore, Li et al. have shown an interesting regulatory pathway that involves SATB1 and NF-*κ*B and exists in breast cancer cells after chemotherapy. According to the cited study, miR-448 suppression (in response to chemotherapy) directly promotes SATB1 expression, which initiates amphiregulin (AR)/EGFR/PI3K/Akt pathway signaling leading to the activation of NF-*κ*B and acquisition by epithelial cells of mesenchymal features (epithelial-to-mesenchymal transition; EMT) [87]. Another regulatory network between SATB1 and NF-*κ*B has been presented by Wang et al. in lymphoblastoid cell lines [88]. In accordance with these reports, we found for the first time that the expression of SATB1 and NF-*κ*B was positively correlated in GC patients. This finding needs to be confirmed by a larger sample size. We also expected to find the correlation between NF-*κ*B and HER2, because the previous report from gastric cancer has demonstrated it and suggested that the expression of these two proteins may play a crucial role in the progression of the disease [89]. Furthermore, breast cancer studies have revealed that NF-*κ*B is downstream of HER2 signaling, and HER2-induced NF-*κ*B activation potentially underlies drug resistance and tumor growth [90–92]. However, we did not find any relationship between NF-*κ*B and HER2 expression in our set of GC cases.

## 5. Conclusion

In conclusion, to the best of our knowledge, there are no reports which simultaneously analyzed the expression of HER2, NF-*κ*B, and SATB1 proteins in patients with diagnosed gastric cancer. In our cohort of patients, HER2 and SATB1 were found to be overexpressed in gastric cancer tissue in comparison to normal gastric mucosa. The expression status of the former protein was seen to differ according to some clinicopathological features, but without statistical significance, whereas the expression of the latter was not importantly associated with any of them. In turn, the NF-*κ*B protein level, which did not differ significantly between GC and noncancerous tissues, was found to be significantly related to the presence of lymph node metastasis. Furthermore, the positive percentage of the NF-*κ*B expression was markedly more common in the intestinal histological type than in the diffuse type; however, there was no difference in NF-*κ*B positivity between the intestinal and mixed types of gastric carcinoma. In the entire cohort, the HER2 expression was not significantly correlated with that of other proteins, but instead, a positive correlation was found between the expression of SATB1 and NF-*κ*B. Further studies with a larger group of patients combined with *in vitro* mechanistic experiments are required to fully elucidate the role and relationship of HER2, NF-*κ*B, and SATB1 expression in gastric cancer progression, as well as to assess the clinical significance of their joint detection in GC tissue samples.

## Figures and Tables

**Table 1:** Clinicopathological properties of 104 patients with gastric carcinoma.

Variables	No. of cases *n* = 104	Percentage (%)
Age (years)		
≤60	27	26.0
>60	77	74.0
Gender		
Male	73	70.2
Female	31	29.8
Lauren's classification		
Intestinal	52	50.0
Diffuse	41	39.4
Mixed	11	10.6
Grading		
G1	2	1.9
G2	45	43.3
G3	57	54.8
pT status		
T0	8	7.7
T1	4	3.8
T2	27	26.0
T3	50	48.1
T4	15	14.4
pN status		
N0	40	38.5
N1	33	31.7
N2	27	26.0
N3	4	3.9
Location		
Cardia	33	31.8
Fundus	38	36.5
Antrum	12	11.5
Pylorus	21	20.2
Tumor size (cm)		
<5	41	39.4
≥5	63	60.6

**Table 2:** Immunoreactivity results for HER2, NF-*κ*B, and SATB1 in association with clinicopathological characteristics of patients with gastric cancer.

Clinicopathological features	*n* (%) *n* = 104	HER2 expression		*P* value	NF-*κ*B expression		*P* value	SATB1 expression		*P* value
		Low *n* = 84	High *n* = 20		Low *n* = 67	High *n* = 37		Low *n* = 72	High *n* = 32	
Age (years)				0.05			0.82			0.33
≤60	27 (25.96)	18 (66.67)	9 (33.33)		18 (66.67)	9 (33.33)		21 (77.78)	6 (22.22)	
>60	77 (74.04)	66 (85.71)	11 (14.29)		49 (63.64)	28 (36.36)		51 (66.23)	26 (33.77)	
Gender				>0.99			0.18			0.82
Male	73 (70.19)	59 (80.82)	14 (19.18)		44 (60.27)	29 (39.73)		51 (69.86)	22 (30.14)	
Female	31 (29.81)	25 (80.65)	6 (19.35)		23 (74.19)	8 (25.81)		21 (67.74)	10 (32.26)	
Lauren's classification				0.63			0.07			0.15
Intestinal	54 (51.92)	42 (77.78)	12 (22.22)		30 (55.56)	24 (44.44)		36 (66.66)	18 (33.33)	
Diffuse	41 (39.42)	35 (85.37)	6 (14.63)		32 (78.05)	9 (21.95)		31 (75.61)	10 (24.39)	
Mixed	9 (8.65)	7 (77.78)	2 (22.22)		5 (55.56)	4 (44.44)		5 (55.55)	4 (44.44)	
Grading				0.23			0.17			0.29
G1	2 (1.92)	0 (0.00)	2 (100.0)		2 (100.0)	0 (0.00)		2 (100.0)	0 (0.00)	
G2	45 (43.27)	37 (82.22)	8 (17.78)		24 (53.33)	21 (46.67)		28 (62.22)	17 (37.78)	
G3	57 (54.81)	47 (82.46)	10 (17.54)		41 (71.93)	16 (28.07)		42 (73.68)	15 (26.32)	
pT status				0.34			0.21			0.92
T0	8 (7.69)	8 (100.0)	0 (0.00)		3 (37.50)	5 (62.50)		6 (75.00)	2 (25.00)	
T1-T2	31 (29.81)	24 (77.41)	7 (22.58)		22 (70.97)	9 (29.03)		21 (67.74)	10 (32.26)	
T3-T4	65 (62.50)	52 (80.00)	13 (20.00)		42 (64.62)	23 (35.38)		45 (69.23)	20 (30.77)	
pN status				0.21			**0.04**			0.66
N0	40 (38.46)	35 (87.50)	5 (12.50)		31 (77.50)	9 (22.50)		29 (72.50)	11 (25.00)	
N1-N3	64 (61.54)	49 (76.56)	15 (23.44)		36 (56.25)	28 (43.75)		43 (67.20)	21 (32.80)	
Location				0.48			0.82			0.88
Cardia	33 (31.73)	26 (78.79)	7 (21.21)		20 (60.61)	13 (39.39)		24 (72.73)	9 (27.27)	
Fundus	38 (36.54)	32 (84.21)	6 (15.79)		25 (65.79)	13 (34.21)		25 (65.79)	13 (34.21)	
Antrum	12 (11.54)	11 (91.67)	1 (8.33)		9 (75.00)	3 (25.00)		9 (75.00)	3 (25.00)	
Pylorus	21 (20.19)	15 (71.43)	6 (28.57)		13 (61.91)	8 (38.09)		14 (66.67)	7 (33.33)	
Tumor size (cm)				0.80			0.53			0.52
<5	41 (39.42)	34 (82.93)	7 (17.07)		28 (68.29)	13 (31.71)		42 (66.67)	21 (33.33)	
≥5	63 (60.58)	50 (79.37)	13 (20.63)		39 (61.90)	24 (38.10)		30 (73.17)	11 (26.83)	

*P* value with statistical significance is marked in bold (Fisher's exact test).

## Data Availability

The data used to support the findings of this study are available from the corresponding author upon request.
